# Ewing family tumors of soft tissue: a case report

**DOI:** 10.11604/pamj.2025.51.55.39626

**Published:** 2025-06-23

**Authors:** Anass Haloui, Nassira Karich, Nada Akouh, Nadir Miry, Rim Ouajdi, Siham Nasri, Larbi Benradi, Mohamed Belahcen, Amal Bennani

**Affiliations:** 1 Laboratory of Pathological Anatomy, Mohammed VI University Hospital, Faculty of Medicine and Pharmacy of Oujda, Mohammed First University, Oujda, Morocco,; 2 Department of Radiology, Mohammed VI University Hospital, Faculty of Medicine and Pharmacy of Oujda, Mohammed First University, Oujda, Morocco,; 3 Department of Pediatric Surgery, Mohammed VI University Hospital, Faculty of Medicine and Pharmacy of Oujda, Mohammed First University, Oujda, Morocco

**Keywords:** Ewing family of tumors, soft tissue, extraskeletal ewing sarcoma, primitive neuroectodermal tumor, case report

## Abstract

The Ewing family tumors (EFT) of soft tissue represent a spectrum of neoplasms of uncertain histogenesis, arising in soft tissue without bone involvement. They include Extraskeletal Ewing Sarcoma and Primitive Neuroectodermal Tumor. These tumors are morphologically indistinguishable from Ewing sarcoma of the skeletal system. Known to be translocation-associated neoplasms, they share a common non-random translocation leading to the fusion of the EWSR1 gene on the 22q12 region, with one of the many members of the ETS family of transcription factors. Occurring mainly in adolescents and young men under the age of 30, EFT´s may arise virtually anywhere, but are most common in deep soft tissues of the extremities. The purpose of this work is to report a rare case of an EFT arising in the upper arm of a 14-year-old boy, who presented with a painful right arm mass evolving for 6 months, while highlighting the main histological, immunohistochemical and molecular features of this rare condition, along with an original flow chart of a relevant diagnostic approach for differential diagnosis.

## Introduction

The Ewing family tumors (EFT) are a spectrum of neoplasms of uncertain histogenesis, comprising Ewing´s sarcoma and Primitive Neuroectodermal Tumor (PNET). They are the second most common malignant bone tumor after osteosarcoma in children and adolescents. In most instances, soft tissue involvement is due to extensions of the tumor originating in the underlying bone. However, in the absence of bone involvement, these are referred to as extraskeletal Ewing's sarcomas (EES) or PNET. Given the compelling evidence suggesting a common neural histogenesis and tumor genetics [1], both tumors are currently classified as members of the EFT. Localization in soft tissues remains rare but possible, and the diagnosis can be challenging since several types of neoplasms may have an identical morphological appearance or even a similar immunohistochemical profile, giving molecular biology techniques an undeniable role in problematic cases. Here we report a case of soft tissue EFT occurring in the upper arm of a 14-year-old boy, while outlining a practical diagnostic approach that allows for a reliable diagnosis.

## Patient and observation

**Patient information:** a 14-year-old boy with no medical or surgical history, consulted for a painful right arm swelling, gradually increasing in size over 6 months.

**Clinical findings:** physical examination found a painful and mobile subcutaneous mass of the right lateral upper arm, of soft consistency.

**Timeline of current episode:** in November 2021, a magnetic resonance imaging (MRI) was performed, revealing a subcutaneous tumoral mass of the right upper arm. A few days later, a biopsy of the mass was carried out.

**Figure 1:**
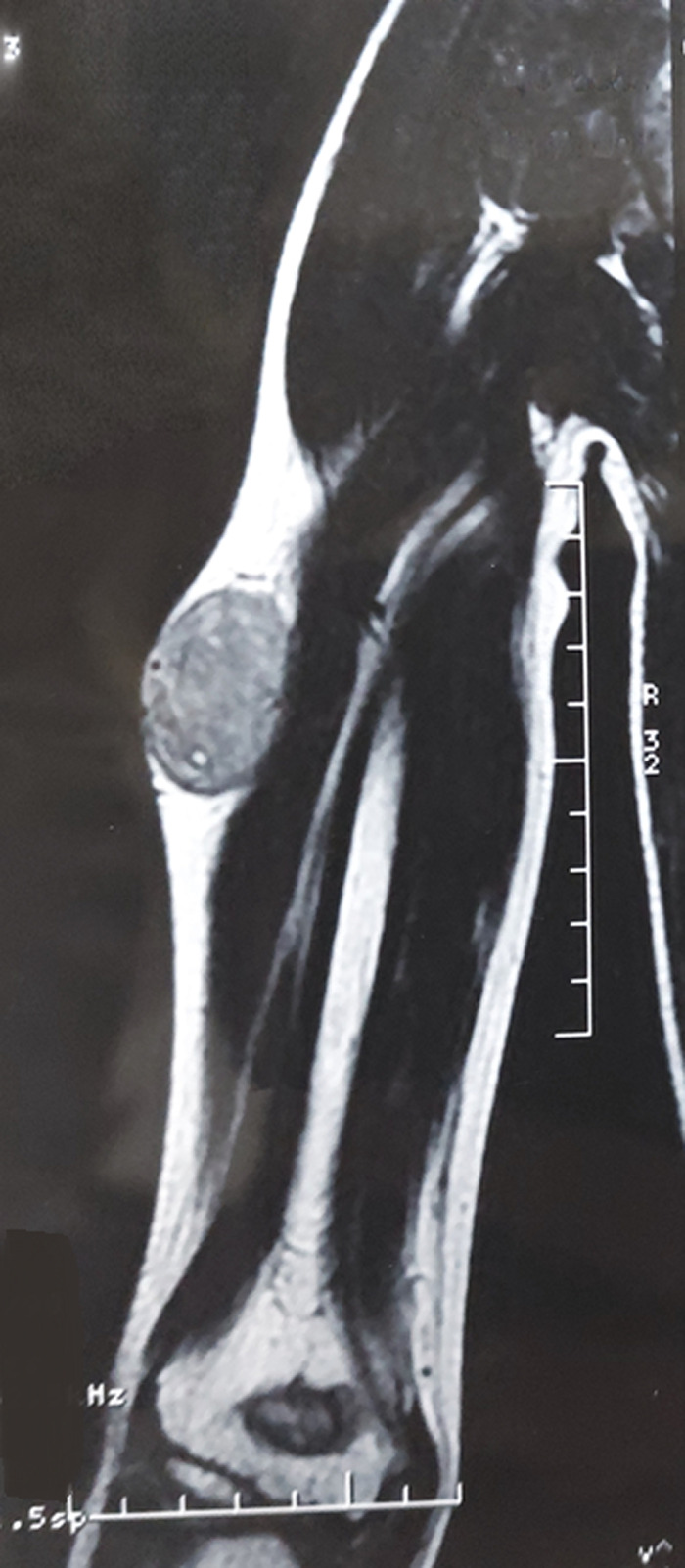
MRI of the right upper arm showing a well-limited subcutaneous mass, surrounded by a pseudo-capsule, without bone involvement

**Figure 2:**
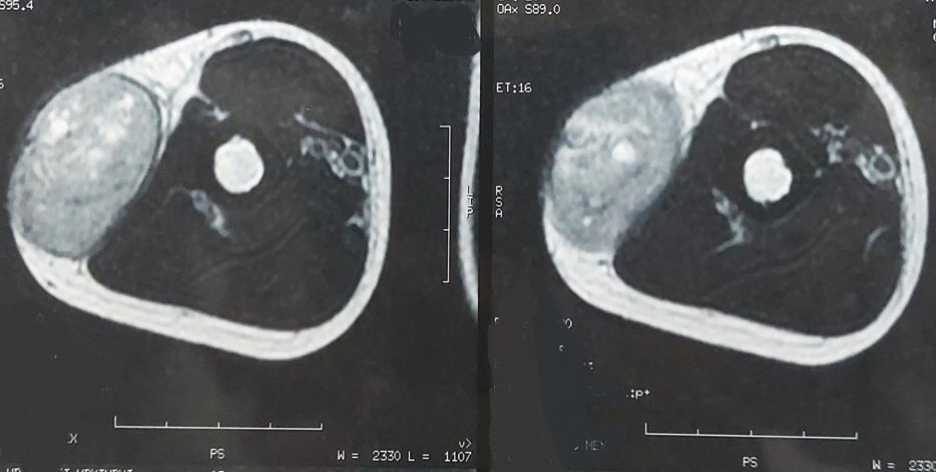
MRI of the right upper arm showing a supra-aponeurotic mass, heterogeneous in appearance, with blurred outlines, in contact with the aponeurosis of the triceps brachii muscle, without bone involvement

**Figure 3:**
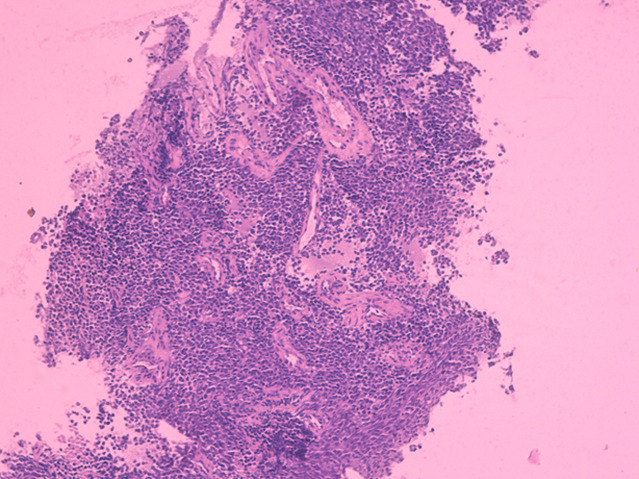
low power view showing a tumoral proliferation made of closely packed monotonous cells, arranged within a richly vascular stroma; hematoxylin and eosin staining, magnification x4)

**Figure 4:**
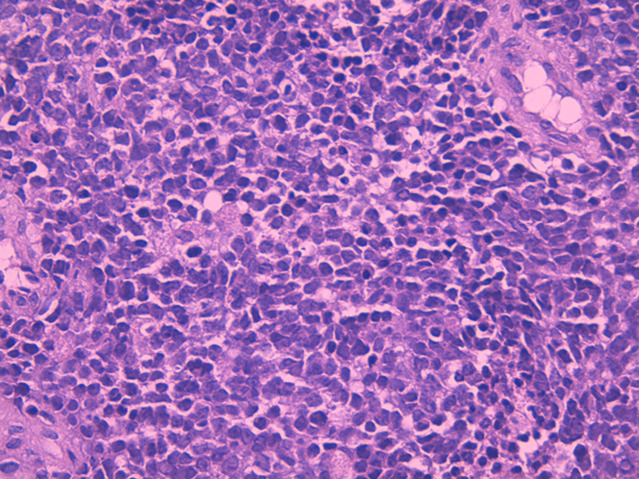
high power view showing tumor cells displaying round nuclei, fine chromatin, inconspicuous nucleoli and scant eosinophilic cytoplasm; hematoxylin and eosin staining, magnification x40)

**Figure 5:**
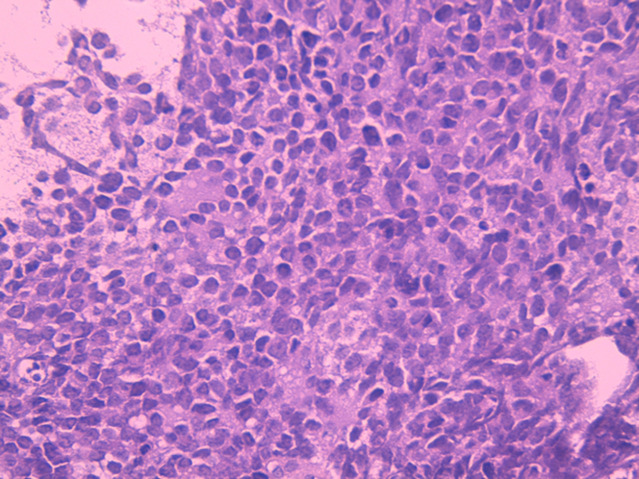
high power view displaying uniformity of tumor cells, with occasional Homer Wright rosettes; hematoxylin and eosin staining, magnification x40)

**Figure 6:**
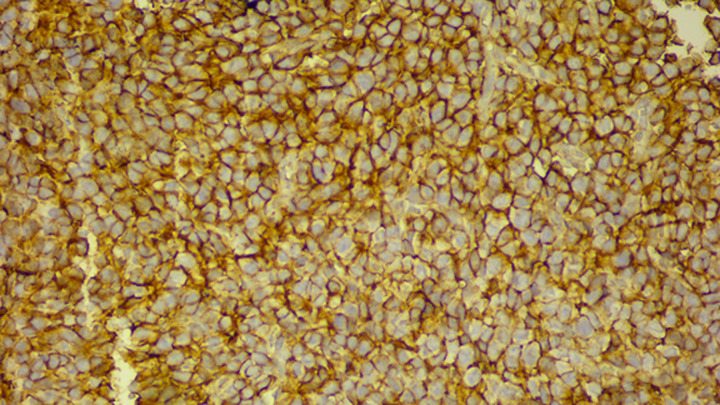
strong and diffuse membranous expression of CD99 by tumoral cells; magnification x20)

**Diagnostic assessment:** the MRI performed revealed a subcutaneous tumoral mass, with irregular seams, measuring 7x5cm, in contact with the aponeurosis of the triceps brachii muscle, without bone, muscle, or radial nerve invasion, in T1 iso-signal and T2 hypersignal (Figure 1, Figure 2). All serum parameters were normal. The biological workup was normal. The histological examination of the biopsy specimen revealed a tumoral proliferation made of sheets of small round blue cells, displaying uniform round nucleus, fine chromatin, inconspicuous nucleoli, and a moderate amount of eosinophilic cytoplasm (Figure 3, Figure 4). Focally, tumor cells were arranged in rosettes structures (Figure 5). The mitotic count was low. Immunohistochemically, tumors cells showed strong and diffuse membranous expression of CD99 (Figure 6), without expression of CD20, CD3, desmin, myogenin, chromogranin A and synaptophysin. A fluorescence in situ hybridization (FISH) study was performed and demonstrated the presence of EWS/FLI1 fusion gene.

**Diagnosis:** the diagnosis of a small round blue cell tumour belonging to Ewing family of soft tissue was retained, based on the histological and immunohistochemical features, which was further endorsed by the evidence of EWS/FLI1 fusion gene by FISH.

**Therapeutic interventions:** the patient underwent neoadjuvant chemotherapy using vincristine, doxorubicin, and cyclophosphamide, before a surgical resection of the tumor mass was performed.

**Follow-up and outcome of interventions:** to date, the patient remains asymptomatic and in excellent clinical condition, with no reported complications.

**Patient perspective:** “I feel well.”

**Informed consent:** informed consent was obtained from the patient.

## Discussion

Localization of EFT in soft tissue remains rare but possible. In 1918, Stout described a case of undifferentiated round cells forming rosettes, within an ulnar nerve tumor in a 42-year-old man [2]. In 1921, Ewing described the first case of round cell neoplasm in the radius of a 14-year-old boy [3]. It was not until 1975 that Angervall and Enzinger reported the first case of extraskeletal Ewing sarcoma arising in soft tissue [4].

EFT mostly affects adolescents and young adults, aged 5 to 30 years, with a slight male predilection [5]. It usually presents as a rapidly growing mass measuring 5 to 10 cm. Other symptoms may include pain and sensory or motor disturbances. They can arise in virtually any anatomic site, but they are more common in deep soft tissues of extremities, with the upper thigh and buttock being the most frequent sites, followed by the upper arm and shoulder. Rarely, the tumor develops in the paravertebral soft tissue or the chest wall [6]. Computed tomography and MRI imaging are useful adjuncts to the diagnosis of EFT. On CT scan, the tumor is usually surrounded by a pseudo capsule, conferring a well-limited character to the mass. It appears hypodense of low attenuation. Although these findings are not specific, they can help differentiate the tumor from the osseous form. Magnetic resonance imaging (MRI) allows better visualization of the anatomical relationships, detection of possible metastasis and evaluation of the therapeutic response to adjuvant therapy [7]. Our case involved a 14-year-old boy who presented a subcutaneous mass of the right arm. The magnetic resonance imaging (MRI) revealed a supra-aponeurotic tumoral mass, heterogeneous in appearance, with blurred outlines, appearing in contact with the aponeurosis of the triceps brachii muscle, without bone involvement, which is consistent with the literature (Figure 1, Figure 2).

On gross examination, the tumor usually appears as multilobulated, soft and friable mass, rarely exceeding 10 cm in size. The cut surface shows a gray-yellow aspect, with possible necrosis, cyst formation and hemorrhage [5]. A relatively wide spectrum of morphologic appearances can be encountered in EFT. The classic Ewing sarcoma is described as a tumor displaying a tightly packed monotonous small cells, with round nuclei, fine chromatin, inconspicuous nucleoli, and scant eosinophilic or clear cytoplasm (due to intracellular deposits of glycogen) [8]. Larger tumoral cells with prominent nucleoli and irregular contour would suggest the diagnosis of an atypical Ewing sarcoma. The presence of Homer-Wright rosettes is more commonly seen in PNET, although it remains a nonspecific finding. Mitotic activity may be prominent. The stroma is usually richly vascular, with numerous thin-walled vessels being compressed by the closely packed tumor cells. Extensive hyalinization of the tumoral stroma can also be found. Rarely, cartilaginous or osseous differentiation may be seen [6]. Numerous studies have attempted to set apart members of EFT on the basis of morphologic criteria, but because these tumors are closely related, the precise criteria for designating a tumor as EES, atypical ES, or PNET remain to date inconclusive. In the past, PNET was diagnosed if the tumor showed well-defined rosettes of Homer Wright or Flexner-Wintersteiner type, if there were at least two positive neural markers or if there was ultrastructural evidence of neural differentiation. Currently, all of these tumors are classified as members of the EFT, regardless of the presence or absence of histological or immunohistochemical evidence of neural differentiation [6]. In our case, the histological findings were consistent with literature, with a tumoral proliferation made of monotonous small round cells, closely arranged in tight packs with few Homer-Wright rosettes, provided with round nucleus, inconspicuous nucleoli, and scant eosinophilic cytoplasm, disposed within a richly vascular stroma (Figure 3, Figure 4, Figure 5).

Immunohistochemically, strong and diffuse membranous expression of CD99 is found in nearly 95% of EFT. Although highly sensitive, CD99 lacks specificity and should be used as part of a panel of immunostains. FLI1 and ERG are often expressed in cases with corresponding gene fusions [9], whereas chromogranin, synaptophysin, CD56 and PS100 are usually negative, although expression of these markers by PNET is possible. In our case, tumor cells showed diffuse and strong membranous expression of CD99 (Figure 6). Whereas lymphoma, rhabdomyosarcoma, small cell carcinoma, desmoplastic small round cell tumor and neuroblastoma were excluded by negative staining for cytokeratin, CD20, CD3, desmin, myogenin, synaptophysin and chromogranin A.

**Figure 7:**
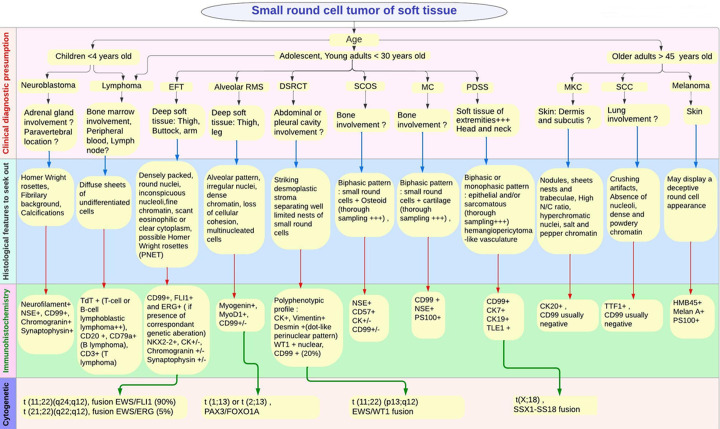
flow chart of a diagnostic approach to small round cell tumors arising in soft tissue/EFT: Ewing family of tumors; RMS: rhabdomyosarcoma; DSRCT: desmoplastic small round cell tumor; SCOS: small cell osteosarcoma; MC: mesenchymal chondrosarcoma; PDSS: poorly differentiated synovial sarcoma; MKC: merkel cell carcinoma; SCC: small cell carcinoma

The genetic hallmark of EFT is the presence of a translocation involving the EWS gene that can be detected by fluorescence in situ hybridization, cytogenetics, or PCR-based methods. The most common translocation (90% of cases) is t(11; 22) (q24; q12), which creates the EWS-FLI1 fusion gene and results in the expression of a chimeric protein. The second most common is t(21;22)(q22;q12) (occurring in 5% of cases), which results in fusion of EWS-ERG fusion gene [8]. The FISH study that was performed for our case revealed the presence of the fusion gene EWS-FLI1, confirming the diagnosis of soft tissue EFT. Numerous other tumors may display an appearance of small round cells leading to misdiagnosis. Figure 7 provides a flowchart of a diagnostic approach covering the main small round cell tumors arising in soft tissue. Table 1 displays the main clinical, histological, immunohistochemical and molecular features of EFT and differentials. Neuroblastoma may simulate an EFT, given the usual young age of patients, the common paravertebral location, and the frequent presence of Homer Wright rosettes. However, they are composed of sheets of small round cells arranged in lobules separated by delicate fibrovascular stroma, which tends to have mats of neuropil. Neuroblastoma usually express neural antigens (NSE, neurofilament protein) and does not express CD99. Alveolar rhabdomyosarcoma may display densely packed cellular areas. However, an alveolar pattern, loss of cellular cohesion and occasional multinucleated giant cells can be found. Some alveolar rhabdomyosarcomas can express CD99, but they also express myogenic markers, including myogenin and MyoD1. Lymphoma can show a deceptive appearance of undifferentiated tumor cells. Therefore, careful seeking of the lobular arrangement and monotonous uniformity of nuclei in EFT can be helpful in making the right diagnosis. It should be kept in mind that a subset of lymphomas, including T-cell lymphoblastic lymphomas can express CD99. The use of a panel including T- and B-cell markers (CD20, CD3, TdT) is usually helpful to avoid a misdiagnosis.

**Table 1:** main clinical, histological, immunohistochemical, and molecular features of small round cell tumors arising in soft tissue

Small round cell tumor	Age of presentation	Frequent localization	Histologic features	Immunohistochemistry / Genetics
EFT	Adolescents and young men under 30 years old	Deep soft tissues of the extremities (upper thigh and buttock+++)	Tightly packed, monotonous, small cells round nuclei, fine chromatin, inconspicuous nucleoli scant eosinophilic or clear cytoplasm Homer Wright rosettes are possible (PNET)	CCD99+, FLI1 + and ERG1+ Usually negative for CD56, Chromogranin, and Synaptophysin. t(11;22)(q24;q12), fusion EWS/FLI1 (90%)
Neuroblastoma	Children, in the first 4 years of life	Most commonly occurs in the abdomen, especially adrenal gland. Arise along the distribution of sympathetic ganglia. Paravertebral location is possible.	Densely packed small round cells in a lobulated pattern Fibrillary background Homer Wright rosettes Dystrophic calcification	Neural antigens +, CD99 - CD56+, Chromogranin+, Synaptophysin + Lack EWSR1 aberrations Deletions at 1p36 and 11q23, gains of 17q MYCN amplification and TrkA expression
Alveolar rhabdomyosarcoma (Solid pattern)	Early to mid-teens but all ages affected	deep soft tissues of the thigh and leg	Solid pattern (Children+): densely packed cellular areas. Nuclei contain more chromatin, tend to be more irregular. Areas with loss of cellular cohesion and alveolar pattern. Multinucleated cells	Myogenin+, MyoD1+,CD99 +/- t(1;13) or t(2;13) PAX3/FOXO1A
Lymphoma (T-cell and B-cell lymphoblastic lymphomas)	Children, teens, and young men	Bone marrow, peripheral blood or lymph nodes soft tissue is rare	Sheet of undifferentiated cells. Lack lobular arrangement	Tdt +, CD99 +/-, LCA +/- CD20 +/-CD79a+/-, CD3 +/-
Desmoplastic small round cell tumor	Young men (Peak incidence in the third decade of life)	Abdominal cavity+ Other: pleural cavity, CNS, Orbit, soft tissue	Well-defined nests of small round cells separated by desmoplastic stroma	Polyphenotypic profile: CK+, Vimentine+, Desmin + WT1 + , CD99 + (20%) t(11;22)(p13;q12) EWSR1-WT1 fusion gene
Metastatic small-cell pulmonary small cell carcinoma	Adults (over 45 years old)	Lung Metastasis to soft tissue may occur	Small round cells Crushing artifacts +	TTF1+ CD99 usually negative
Merkel cell carcinoma	Adults (over 60 years old)	Skin: in the Dermis and subcutis.	Large cells, closely packed nuclei and little cytoplasm, trabecular pattern	CK20+, CD99 usually negative
Mesenchymal chondrosarcoma	Young adults	Extraosseous 20%	biphasic appearance: nodules of well-differentiated cartilage admixed with undifferentiated areas of round cells	CD99 +, NSE+, PS100+ Lack EWSR1 aberrations
Small cell osteosarcoma	Young age	Rarely extraosseous	Small round cells + osteoid	NSE+, CD57+, CK+/-, CD99+/- Lack EWSR1 aberrations
Poorly differentiated synovial sarcoma	Adolescents and young adults	Soft tissue of extremities+ Head and neck	small round cells often arranged around a hemangiopericytoma-like vasculature. biphasic or monophasic pattern	CD99+, CK7+, CK19+, TLE1 + t(X;18), SSX1-SS18 fusion gene
Melanoma	Old patient	skin	May display a deceptive round cell appearance	HMB45+ Melan A+ PS100+

Desmoplastic small round cell tumor is a highly aggressive neoplasm, typically occurring in young men. It is usually composed of well limited nests of small round cells separated by a desmoplastic stroma. However, the localization is usually intra-abdominal, although soft tissue localization is possible. Even though these tumors can express CD99, they tend to have a polyphenotypic profile expressing CK, Vimentin, desmin with a unique perinuclear pattern of staining, NSE and nuclear expression of WT1. Furthermore, they are characterized by a unique translocation t(11;22)(p13;q12) involving the WT1 gene on 11p13 and EWSR1 gene on 22q12 [10]. Metastatic pulmonary small cell carcinoma can be ruled out by positivity of TTF1, which is absent in EFT. Merkel cell carcinoma tends to occur in much older patients, usually over 60 years old, and they tend to express CK20, a marker generally negative in EFT. Mesenchymal chondrosarcoma is characterized by a biphasic appearance of nodules of well-differentiated cartilage admixed with undifferentiated areas of round cells. This characteristic biphasic appearance may not be seen on biopsy specimen, which can be a potential pitfall resulting in a misdiagnosis of EFT. Furthermore, the majority of mesenchymal chondrosarcomas show strong membranous CD99 immunoreactivity. However, they lack EWS aberrations. Small cell osteosarcoma typically occurs in young patients and is composed of cells similar to those seen in EFT. The presence of osteoid which is required for the diagnosis may be only focally present in the tumor, and is often not identified in small biopsy specimens, making distinction from EFT difficult. They rarely express CD99, and they lack EWS aberrations as well. Poorly differentiated synovial sarcomas are composed of small round cells often arranged around a hemangiopericytoma-like vasculature. Unless other areas of biphasic or monophasic synovial sarcoma are seen, and because they usually show membranous CD99 immunoreactivity, immunohistochemical distinction of poorly differentiated synovial sarcoma from EFT may be difficult. CK 7 and 19 can be used to distinguish the two tumors, as they are often positive in poorly differentiated synovial sarcomas and are rarely, if ever, expressed in EFT. In addition, almost all cases of synovial sarcomas, including poorly differentiated tumors, show strong nuclear expression of TLE1, a marker that is not present in EFT. However, detection of t(X;18) by conventional cytogenetics or the resulting SSX1-SS18 or SSX2-SS18 fusion by molecular techniques is an important diagnostic aid in these cases as well (Table 1).

## Conclusion

Given the wide variety of small round cell tumors that can arise in soft tissue, the diagnosis of EFT must be methodically conducted, with a careful morphological examination and cautious interpretation of immunostains. Confirmation by molecular work-up remains the best approach for a reliable diagnosis in challenging cases.
